# AI-driven financial risk management in complex mobile economies: a contextual-technology fit and security reassurance model

**DOI:** 10.3389/frai.2026.1888236

**Published:** 2026-06-15

**Authors:** Ziwen Jiang, Xu Jiang

**Affiliations:** 1 XuBeihong Art Academy, Shanghai Maritime University, Shanghai, China; 2 Academy of Arts & Design, Tsinghua University, Beijing, China

**Keywords:** complex financial systems, contextual-technology fit, reassurance confirmation, security, transaction risk management

## Abstract

The rapid evolution of artificial intelligence in financial technologies has fundamentally reshaped risk management within complex socio-economic systems. However understanding how users build transactional trust within high-density mobile economies remains a critical challenge. This study integrates technology fit and reassurance confirmation theories to investigate user security perceptions in complex micro-economic environments. A field survey was conducted aboard a mega cruise vessel yielding 329 valid responses that were analyzed through structural equation modeling. The empirical findings reveal that contextual technology fit significantly improves secured sensory interaction and perceived architectural quality. Perceived architectural quality acts as a vital structural intermediary that updates security expectations and enhances reassurance confirmation. Ultimately reassurance confirmation and secured sensory interaction are identified as the primary drivers of overall trust and satisfaction. This research provides a significant conceptual advance by shifting the narrative of technology acceptance from simple functional efficiency to a comprehensive framework encompassing spatial safety and micro-financial risk perception. The conclusions offer actionable strategies for system operators to leverage artificial intelligence interfaces for regulatory compliance and systemic security control in modern financial ecosystems.

## 1 Introduction

While artificial intelligence significantly optimizes transactional efficiency in modern micro-economies, it simultaneously exposes these complex networks to novel financial risks and governance difficulties. Modern mega cruise liners have transformed from simple marine transport vessels into isolated and highly dense economies that handle real-time transactional tracking and high-frequency offline-to-online commerce. Within these specialized closed-loop systems, passengers face concrete digital financial transaction risks. Specifically, when offshore network connectivity is restricted, offline QR-code transactions suffer from synchronization delays, which directly triggers double-payment fraud vulnerabilities. Furthermore, the complex three-dimensional, multi-deck layouts cause severe navigational confusion, generating user privacy leakage anxieties during micro-payment interface operations in crowded areas. Traditional two-dimensional signage and standard automated tools frequently fail to address these situational threats and informational asymmetries. Therefore, an optimized artificial intelligence guidance platform must be implemented to provide real-time path steering and data-transparent interaction, serving as a proactive micro-risk reduction component engineered to preserve systemic safety and construct transactional trust.

The theoretical baseline for interpreting user behavioral acceptance and continuous compliance within such specialized environments is profoundly rooted in the Task-Technology Fit framework. This structural model establishes that the operational efficacy and long-term adherence of an advanced digital system rely heavily on the explicit compatibility between the functional attributes of the technology and the specific task requirements of the individual user (Goodhue and Thompson, 1995). In safety-critical or isolated maritime environments, this configuration requires a robust contextual-technology fit, where the localized artificial intelligence system adapts its information architecture to clear environmental risk constraints and transactional security needs. Utilizing complex digital interfaces for international transit environments demonstrates that a high degree of technology fit can systematically eliminate uncertainty during high-stakes information seeking and decision making (D’Ambra and Rice, 2004). Simultaneously, the psychological mechanism of user retention and system trust is governed by the Expectation Confirmation Model, which posits that post-usage satisfaction is a dynamic cognitive adjustment derived from whether actual system performance successfully confirms initial expectations (Bhattacherjee, 2001).

While digital infrastructures are increasingly integrated into smart transit architectures, empirical research examining how automated user interfaces neutralize psychological anxiety and forge baseline structural trust remains restricted. Merging the principles of technology fit and expectation confirmation into a unified framework provides an advanced perspective for explaining individual continuous behavioral patterns and performance outcomes in complex online environments (Ayyoub et al., 2023). This study systematically addresses these theoretical and structural gaps by utilizing China’s first independently constructed mega cruise liner, the Adora Magic City, as a specialized empirical setting for complex micro-economies. By merging the principles of Contextual-Technology Fit and Reassurance Confirmation into a unified structural equation model, this inquiry investigates how the structural order and interactive clarity of an artificial intelligence guidance gateway translate into psychological certainty for the user. The findings move beyond basic acceptance metrics to articulate the precise secured sensory interaction pathways that eliminate operational friction, manage micro-financial risks, and secure long-term continuous usage intentions.

## 2 Literature review

### 2.1 Theoretical foundations of contextual-technology fit in complex systems

The structural integration of digital platforms into complex environments necessitates an explicit alignment between structural system functionalities and the situational demands placed upon the user. Within information systems analysis, the core blueprint for investigating this relationship is established by the Task-Technology Fit (TTF) framework. Introduced fundamentally by Goodhue and Thompson (1995), the framework asserts that individual performance outcomes and technological utilization rates are heavily determined by the degree of compatibility between the capabilities of the digital tool and the specific task requirements of the actor. Prior to the formulation of this fit paradigm, early researchers predominantly evaluated system success through generalized, subjective user evaluations, focusing primarily on information accuracy and transactional timeliness (Bailey and Pearson, 1983). Other foundational adoption frameworks, such as the Technology Acceptance Model (TAM), isolated perceived usefulness and perceived ease of use as the primary cognitive precursors to adoption behavior (Davis et al., 1989). While these early conceptual models contributed significantly to understanding initial technology acceptance, they frequently overlooked the critical intersection between situational operational constraints and technological adaptability (Dishaw and Strong, 1999).

The development of precise evaluation measures for emerging digital applications across specialized industries required a broader analytical focus on task-technology alignment to guarantee absolute structural integrity (Benbasat and Moore, 1992). The fit model systematically addressed these theoretical limitations by introducing the explicit concept of compatibility between situational task characteristics and specific technological features. Subsequent empirical validation demonstrated that information systems achieve optimal individual and organizational performance only when user capabilities, operational tasks, and technological attributes are aligned simultaneously (Goodhue and Thompson, 1995). In regulatory or highly enclosed environments where technology utilization becomes mandatory for navigation or transaction processing, systemic performance and user compliance are more robustly predicted by the actual degree of fit rather than by basic usage frequency (Goodhue, 1998). Conversely, when an operational mismatch occurs and the digital platform fails to adequately support users during complex workflows, increased usage intensity can actively hinder task performance, introducing severe operational vulnerabilities and structural errors into the system.

As complex spaces evolve into smart socio-economic environments, the specific nature of the “task” changes fundamentally, moving from basic manual operations to highly complex information tracking and real-time transaction processing. In contemporary smart tourism ecosystems, intelligent wayfinding and guidance systems serve as a core technological infrastructure that directly dictates user mobility, safety compliance, and financial service engagement. Despite the growing operational relevance of these localized AI systems, empirical investigations examining their structural adoption behaviors within enclosed maritime frameworks remain sparse (Koh et al., 2023). Early travel and information systems literature highlighted that utilizing complex digital interfaces for international transit demands a high degree of technology fit to systematically eliminate uncertainty during high-stakes information seeking (D’Ambra and Rice, 2004). Similarly, within innovation-driven corporate environments, the strict alignment between business analytical capabilities and situational organizational tasks is recognized as a vital prerequisite for maintaining high search efficiency and preventing strategic execution errors (Muchenje et al., 2024). These diverse empirical applications validate the cross-disciplinary versatility of the fit paradigm across various resource management and automated decision-making contexts (Ye, 2008).

The contemporary integration of fit constructs with distinct psychological and behavioral adoption theories has consistently demonstrated enhanced explanatory power across specialized domains. For instance, empirical research on mobile banking systems revealed that long-term user acceptance is heavily mediated by the perceived compatibility of the technology with the high-security demands of daily financial transactions (Zhou et al., 2009). Furthermore, research examining social media platform adoption among diverse demographic cohorts highlighted that the interaction between perceived systemic risks and the transparency of digital interfaces dictates user retention and data security compliance (Liu, 2015). These varied applications underscore the theoretical flexibility of the fit framework in modeling human-computer interactions within complex markets (Cao, 2013; Zigurs and Buckland, 1998).

Within a modern mega cruise liner, the enclosed spatial morphology, coupled with high-density compartments and a multi-sector commercial infrastructure, creates an unprecedented set of operational micro-risks. The passenger’s task within this specialized environment is inherently dualistic, requiring the simultaneous execution of complex physical navigation and high-frequency real-time financial consumption. Unlike generic urban navigation systems where simple functional efficiency is the singular metric of performance, an isolated maritime guidance system must establish a comprehensive “Contextual-Technology Fit” (RK). This fit measures how effectively the localized AI platform adapts its information delivery to the spatial constraints and transaction risks of the vessel. Extending the fit paradigm into this specialized environment allows researchers to investigate the precise structural determinants of initial acceptance, continuous compliance, and risk minimization within large-scale socio-economic systems.

### 2.2 Psychological mechanisms of perceived architectural quality and security reassurance

The long-term safety and operational stability of complex systems heavily depend on the psychological mechanisms that govern how users evaluate technological performance over time. Expectation Confirmation Theory (ECT), originally developed within consumer behavior literature, states that user satisfaction is a post-consumption evaluation derived from a comparison between actual performance and initial expectations (Oliver, 1981). Oliver established that the consistency between pre-consumption benchmarks and actual outcomes is the primary determinant of post-consumption cognitive adjustments. Alternative theoretical iterations suggested that this evaluation process involves a comparative assessment between current experiences and alternative systemic outcomes. Churchill and Surprenant (1982) expanded the empirical boundaries of confirmation theory by identifying a four-factor structural framework for understanding satisfaction, isolating initial expectations, perceived performance, confirmation level, and overall satisfaction as distinct constructs. Critically, they noted that perceived performance can independently enhance satisfaction even in the absolute absence of a baseline expectation difference, stressing the need to optimize actual performance over mere marketing communication.

The application of expectation confirmation frameworks to the domain of information systems marked a theoretical shift toward post-usage behavioral variables and long-term user retention (Patterson et al., 1997). Introducing complex evaluative parameters, such as customer-perceived system quality and secure value realization, allowed researchers to map user behaviors within highly specialized professional services. Building directly upon these post-consumption insights, Bhattacherjee (2001) formally introduced the Expectation Confirmation Model (ECM) to explicitly evaluate continuous user engagement with electronic transaction systems. The ECM reoriented information systems research by positioning confirmation, perceived usefulness, and post-usage satisfaction as the primary structural determinants of digital service continuance. Within this model, satisfaction is confirmed as the primary cognitive driver that exerts the most significant direct path influence on a user’s intention to remain within a specific digital platform, providing a robust empirical baseline for studying high-security electronic environments where long-term retention is far more critical than initial adoption.

Within the modern domain of automated navigation and smart financial technologies, expectation confirmation frameworks provide an established conceptual lens for interpreting how users form complex security judgments and sustain compliance across extended temporal horizons (Lin et al., 2005). Integrating variables such as interactive playfulness and sensory quality into the confirmation model revealed that emotional enjoyment can actively modify the confirmation process. Specifically, by examining the empirical degree of confirmation after a user has actively interacted with an intelligent guidance interface, researchers can accurately predict system continuance intentions (Hsu et al., 2006). Predicting system adherence within advanced electronic environments frequently requires a decomposed analysis of planned behavior to fully capture the nuances of user intention and risk aversion (Hsu and Chiu, 2004). Furthermore, longitudinal empirical tracking indicates that a user’s post-adoption beliefs are highly dynamic, continuously evolving as they accumulate operational experience with the digital infrastructure. These beliefs are shaped by the exact degree to which the actual system performance aligns with initial expectations regarding transaction safety and structural reliability.

Continuous user engagement within online service platforms is the result of a multi-stage cognitive process where initial confirmation leads to a long-term trust commitment (Kang et al., 2009). The structural integration of task-technology fit theories with expectation confirmation frameworks has emerged as a compelling theoretical approach for designing systems in highly complex scenarios (Ayyoub et al., 2023). This cross-theoretical synthesis explains complex patterns of online interaction and individual performance by simultaneously focusing on functional engineering alignment and post-usage psychological confirmation. This integrated perspective is particularly relevant for high-density social networks and interactive spaces where continuous user retention depends heavily on the ongoing confirmation of safe social and financial value. In health applications and safety-critical digital spaces, users remain actively engaged only when the system provides high functional value for their specific risk-mitigation tasks.

For users navigating the intricate operational networks of a modern mega cruise ship, overall satisfaction is increasingly derived from a specialized mechanism termed “Reassurance Confirmation” (QC). Passengers enter this isolated micro-economy with high pre-consumption security expectations regarding data privacy, transaction compliance, and spatial safety (Oliver, 1981). Consequently, their cognitive evaluation is heavily anchored in “Perceived Architectural Quality” (JX), which measures the structural clarity, visual order, and professional integrity of the AI interface. If the architectural design of the AI interface exhibits a high level of professional integrity, it acts as a powerful security signal that confirms positive user expectations and minimizes perceived fraud vulnerabilities within the complex environment (Lin et al., 2005).

### 2.3 Environmental risks and secured sensory interaction in specialized spaces

Modern specialized environments represent complex spatial and economic systems where traditional, static guidance methods are completely inadequate. In high-density compartments such as those found on advanced cruise vessels, automated wayfinding infrastructures serve as crucial environmental design elements that dictate human traffic flow, reduce physical crowding risks, and secure localized commerce. Wayfinding designs across international transit infrastructures demonstrate that a clear, culturally resonant, and structurally logical signage system significantly improves user environmental perception and orientational certainty. Developing robust empirical instruments for measuring user-perceived quality is an essential requirement for validating these architectural and safety dimensions in digital interfaces (Aladwani and Palvia, 2002). High system quality and structural clarity can lead users to experience a psychological state of flow, characterized by deep immersion, optimal decision-making, and a complete reduction in technological confusion (Dai and Liu, 2015).

This interactive state of flow is closely related to long-term system adherence and continuous service compliance. The structural intention to continue utilizing a specific digital platform is heavily shaped by the user’s systemic dependency on that platform to fulfill core information and safety needs (Chang et al., 2017). Within contemporary mobile media environments, this continuous adherence is further shaped by the perceived quality of visual search, interaction fluency, and user experience within the system interface (Meng and Zhu, 2022). Systematically reducing psychological barriers and cognitive friction is an essential managerial priority for maintaining safe human traffic flow within high-density architectural compartments (Zhan and Yan, 2014). Providing automated navigation experiences that are structurally rich in clear context-specific signs can actively assist users in overcoming these cognitive barriers.

Furthermore, the temporal evolution of individual technological beliefs is profoundly dictated by the exact intensity and frequency of actual system usage over time (Karahanna et al., 1999). For individuals who interact with an intelligent guidance system repeatedly across a multi-day timeline, their subjective perceptions of systemic efficacy and transaction security are continuously modified based on usage intensity. The adoption of emerging AI technologies in complex spaces poses a unique set of structural challenges that require specialized design frameworks (Koh et al., 2023). For example, empirical research on urban drone adoption demonstrates how strict environmental constraints and localized spatial layouts directly influence individual technological acceptance and risk aversion. On a modern mega-liner, the high-density enclosed layout requires automated guidance platforms that are adaptive to user needs at different stages of a journey (Jiang et al., 2023). Making these systems adaptive enhances the user experience and ensures behavioral compliance by delivering highly relevant, risk-optimized information during critical situational shifts.

Navigating complex physical configurations requires automated systems that incorporate spatial topology variables to maintain a completely consistent operational logic (Zhang P. et al., 2019). Research on user-centered digital art and interaction design within public spaces emphasizes that context-specific structural embeddedness is a foundational requirement for securing effective engagement (Jiang et al., 2025). This approach ensures that the digital infrastructure serves as an organic, reliable element of the spatial and socio-economic environment. Aligning localized technological capabilities with broader operational goals ensures that the digital infrastructure actively supports the entity’s overall brand safety and risk management strategy (Pillai and Sivathanu, 2020). For advanced service operators, the ultimate objective is to provide a seamless, highly secure experience that reinforces system equity and user trust. This requires a precise alignment between automated functionalities and the user’s practical workflow requirements.

Operational problem resolution with digital systems is maximized only when the structural features of the technology support the user’s task goals. Long-term habitual behaviors further reinforce the structural relationship between intention and actual usage over extended temporal horizons (Cheung and Lee, 2005). To model the temporal utility and safety performance of these systems in complex modern environments, researchers strongly advocate for the implementation of longitudinal tracking designs (Tam et al., 2020). Designing automated platforms based on these integrated fit and confirmation principles systematically enhances user satisfaction and reinforces systemic security through clear structural communication.

The empirical adoption of healthcare devices confirms that perceived functional performance is a critical factor for long-term engagement in safety-critical contexts (Wang et al., 2020). For intelligent AI guidance systems, this performance is measured by how effectively the system presents real-time navigation cues, spatial layouts, and transaction safety protocols. If the guidance interface is perceived as functional, secure, and structurally cohesive, users are significantly more likely to confirm their positive security expectations. By focusing explicitly on Contextual-Technology Fit and Reassurance Confirmation, complex socio-economic entities can develop advanced digital tools that are optimized for the high-density, high-risk environments of modern automated vessels. This integrated cross-theoretical approach provides actionable insights for developing smart system solutions that contribute to the sustainable advancement and operational safety of the global tourism and transit sectors.

### 2.4 AI-enabled financial security and interface reassurance in micro-payment scenarios

Recent research on AI-enabled financial services suggests that artificial intelligence reshapes user trust not only through back-end risk-control algorithms, but also through front-end interface interactions that make financial security perceptible during use. In mobile banking, digital wallets, and AI-based FinTech services, users rarely observe encryption mechanisms, fraud-detection models, transaction-monitoring infrastructures, or algorithmic risk scoring directly. Instead, they infer the security and reliability of a financial system from visible and interactive cues, including transaction-status transparency, authentication feedback, response speed, error-recovery messages, security prompts, and the perceived professionalism of the interface architecture. Therefore, perceived security in micro-payment scenarios should be understood not merely as a technical property of the financial system, but also as an interface-mediated psychological outcome.

In AI-supported mobile banking contexts, trust and perceived security have been identified as central factors shaping users’ attitudes toward intelligent financial services. Lopes et al. found that perceived service quality and security are important antecedents of trust in AI-enabled mobile banking, and that trust further shapes users’ attitudes toward AI financial services (Lopes et al., 2025). Similarly, Ikhsan et al. examined AI adoption in the Indonesian banking sector by extending the Technology Acceptance Model and incorporating perceived trust, showing that users’ continuance intention toward AI banking services is closely connected with trust formation and perceived technological reliability (Ikhsan et al., 2025). These findings indicate that users evaluate AI financial services not only according to functional efficiency or convenience, but also according to whether the system can provide a stable, secure, and psychologically reassuring interaction experience.

This issue becomes more critical in micro-payment scenarios, where transactions are small in value but high in frequency. Unlike large financial operations, micro-payments often occur under limited attention, time pressure, spatial congestion, and fragmented decision-making. In these conditions, users may not engage in deliberate risk assessment; instead, they rely on immediate interface cues to judge whether a transaction is safe, synchronized, and institutionally protected. Research on mobile banking app adoption has shown that security, risk, institutional trust, and technology trust should be integrated into adoption models because users’ willingness to use financial applications depends heavily on whether the interface and system environment are perceived as trustworthy (Apau et al., 2025). From this perspective, micro-payment security is not only a matter of encryption or fraud prevention, but also a matter of how effectively the system communicates security to users during the payment process.

AI-based FinTech research further demonstrates that transparency, privacy concerns, and security concerns directly influence users’ perceived value and adoption attitudes. Park and Yoon argued that perceived transparency, perceived responsibility, privacy concerns, and security concerns are key factors affecting the acceptance of AI-based sustainable FinTech tools (Park and Yoon, 2025). Their findings are particularly relevant to AI-guided payment interfaces because users’ trust depends on whether the system makes financial processes understandable and controllable. When transaction feedback is delayed, payment status is ambiguous, or authentication prompts are poorly designed, users may experience anxiety regarding duplicate payment, unauthorized deduction, data leakage, or system failure. Therefore, transparent and interpretable interface feedback becomes a critical mechanism for converting hidden AI risk-control operations into user-perceived security.

The perceived risk literature in FinTech adoption supports this argument. Zhao and Khaliq emphasized that perceived risks remain a major barrier to users’ intention to use FinTech services, even as digital financial services become increasingly widespread (Zhao and Khaliq, 2024). Appiah and Agblewornu further showed that FinTech adoption is shaped by the interaction among perceived benefits, perceived risks, and trust, suggesting that users do not evaluate digital finance only through utility, but through a risk–benefit balance mediated by trust (Appiah and Agblewornu, 2025). Wei et al. similarly demonstrated that mobile FinTech adoption is affected by both perceived value and perceived risk, confirming that privacy and security concerns continue to influence user decision-making in mobile financial environments (Wei et al., 2025). Together, these studies indicate that AI financial interfaces must actively reduce risk perception while simultaneously strengthening the perceived value of secure and convenient transactions.

AI-driven fraud detection research also highlights the importance of trust, transparency, and explainability. Yaseen and Al-Amarneh found that the adoption of AI-driven fraud detection in financial institutions is influenced by trust, transparency, and fairness perception (Yaseen and Al-Amarneh, 2025). Although fraud detection algorithms can improve anomaly recognition and transaction monitoring, opaque algorithmic processes may weaken acceptance if users or institutions cannot understand how risk decisions are generated. In micro-payment environments, this implies that AI security functions should not remain invisible. Instead, risk-control intelligence should be translated into recognizable interface signals, such as clear confirmation messages, payment-status indicators, adaptive warnings, and explainable security prompts. These signals allow users to perceive that the transaction is being monitored, protected, and properly authorized.

This logic is particularly relevant to closed-loop mobile economies such as mega cruise vessels. In such environments, passengers conduct high-frequency consumption and QR-code micro-payments while simultaneously navigating dense, multi-deck, and semi-isolated spatial conditions. Offshore network instability, synchronization delays, crowded payment areas, unfamiliar service routes, and limited environmental knowledge can amplify anxieties about double payment, privacy exposure, transaction failure, or unauthorized deduction. The AI guidance interface therefore functions as a security reassurance gateway: it translates hidden technical safeguards into visible, interpretable, and confidence-building interaction signals. In this study, Secured Sensory Interaction captures the user’s experience of smooth, transparent, and low-friction interaction with the AI system, while Perceived Architectural Quality captures the professional organization, visual order, and structural credibility of the interface. Together, these two constructs explain how Contextual-Technology Fit is translated into Reassurance Confirmation and ultimately into Trust-Satisfaction.

This positioning is also consistent with recent research published in Frontiers in Artificial Intelligence – AI in Finance, where artificial intelligence has been examined as a core mechanism for FinTech risk management, fraud detection, systemic risk monitoring, cyber-risk identification, and trustworthy financial decision-making. Giudici (2018) argues that AI and machine learning technologies can strengthen FinTech risk management by improving fraud detection, systemic risk monitoring, market-risk assessment, and operational cyber-risk identification. Fritz-Morgenthal et al. (2022) further emphasize that financial risk management increasingly requires explainable, trustworthy, and responsible AI because users, institutions, and regulators must understand how AI-supported risk decisions are generated. More recently, Theodorakopoulos et al. (2025) show that AI-powered risk analytics can identify subtle anomalies and weak signals associated with fraud, credit deterioration, and liquidity shortfalls. Building on this line of AI-finance research, the present study shifts attention from institution-facing risk analytics to user-facing security reassurance in micro-payment environments, showing how AI-enabled interfaces translate hidden risk-control mechanisms into perceptible signals of transaction safety, trust, and satisfaction.

Recent AI-finance research has examined artificial intelligence as a core mechanism for FinTech risk management, fraud detection, systemic risk monitoring, cyber-risk identification, and trustworthy financial decision-making. Giudici (2018) argues that AI and machine learning technologies can strengthen FinTech risk management by improving fraud detection, systemic risk monitoring, market-risk assessment, and operational cyber-risk identification. Fritz-Morgenthal et al. (2022) further emphasize that financial risk management increasingly requires explainable, trustworthy, and responsible AI because users, institutions, and regulators must understand how AI-supported risk decisions are generated. More recently, Theodorakopoulos et al. (2025) show that AI-powered risk analytics can identify subtle anomalies and weak signals associated with fraud, credit deterioration, and liquidity shortfalls. Building on this line of AI-finance research, the present study shifts attention from institution-facing risk analytics to user-facing security reassurance in micro-payment environments, showing how AI-enabled interfaces translate hidden risk-control mechanisms into perceptible signals of transaction safety, trust, and satisfaction.

## 3 Research methodology

### 3.1 Research hypotheses

**Figure 1:**
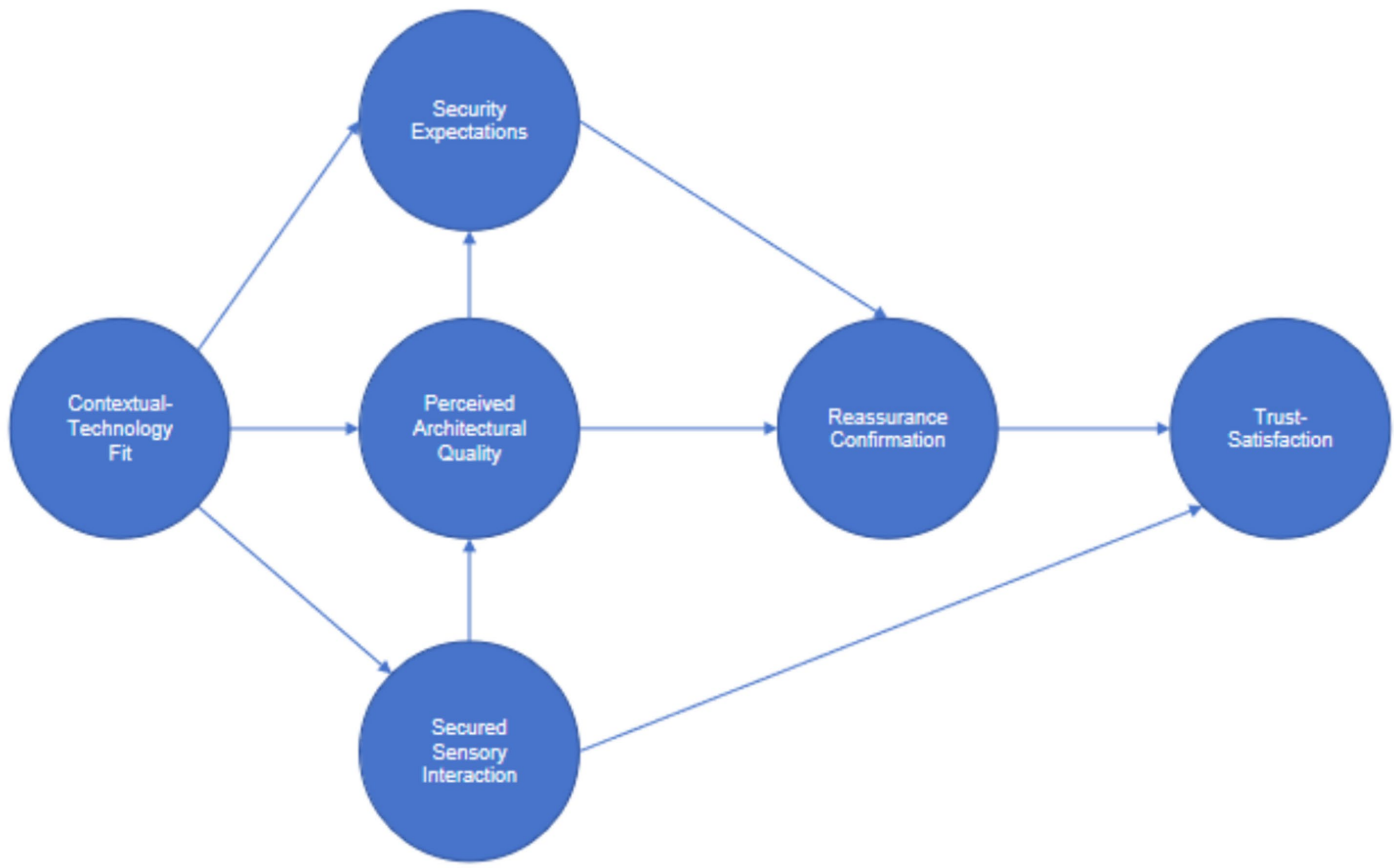
Conceptual model of contextual-technology fit and security reassurance in AI-enabled micro-payment environments.

Drawing on the conceptual model, a set of testable propositions is formulated below, with the structural linkages among them depicted in Figure 1.

*H1*: Contextual-Technology Fit positively influences Security Expectations.

*H1a*: Contextual-Technology Fit positively influences Perceived Architectural Quality.

*H1b*: Contextual-Technology Fit positively influences Secured Sensory Interaction.

*H2*: Security Expectations positively influence Reassurance Confirmation.

*H3*: Perceived Architectural Quality positively influences Reassurance Confirmation.

*H3a*: Perceived Architectural Quality positively influences Security Expectations.

*H4*: Secured Sensory Interaction positively influences Perceived Architectural Quality.

*H4a*: Secured Sensory Interaction positively influences Trust-Satisfaction.

*H5*: Reassurance Confirmation positively influences Trust-Satisfaction.

### 3.2 Questionnaire design

A structured survey instrument was employed in this research. The initial section was designed to obtain background characteristics of the participants (Jiang et al., 2025). The second part focuses on the six main constructs of the research hypotheses: Contextual-Technology Fit, Security Expectations, Reassurance Confirmation level, Perceived Architectural Quality, Secured Sensory Interaction, and Trust-Satisfaction. The second part consists of 22 items, As shown in the Table 1. The items for Contextual-Technology Fit were adapted from Muchenje et al. (2024), Jiang et al. (2023), Koh et al. (2023), Wang et al. (2020), and Liu (2015). The items for Security Expectations and Reassurance Confirmation level were adapted from Lin et al. (2005), Hsu et al. (2006), Kang et al. (2009), Dai and Liu (2015), and Zhan and Yan (2014). The items for Perceived Architectural Quality were adapted from Cao (2013), Karahanna et al. (1999), Aladwani and Palvia (2002). The items for Secured Sensory Interaction were adapted from Chang & Fei (Chang et al., 2017), and Meng and Zhu (2022). The items for Trust-Satisfaction were adapted from Oliver (1981), Churchill and Surprenant (1982), and Patterson et al. (1997). The questionnaire employs a 7-point Likert scale (1 = “Strongly Disagree” to 7 = “Strongly Agree”).

**Table 1:** Measurement items for the six latent constructs and their adapted literature sources.

Latent Variable	Item Code	Measurement Statements (7-point Likert Scale)	Adapted Literature Sources
RK	RK1	The AI guidance system provides real-time transaction information that precisely matches my current location on the cruise decks.	Muchenje et al. (2024), Jiang et al. (2023), Koh et al. (2023), Wang et al. (2020), and Liu (2015)
	RK2	The information structure of this AI platform adapts well to the isolated maritime network environment.	
	RK3	The functional features of the AI system are highly compatible with my on-board micro-payment verification tasks.	
	RK4	The platform offers sufficient navigation cues to support my financial service engagement in crowded spaces.	
	RK5	Overall, the capabilities of this AI system fit the spatial constraints and transactional security needs of the vessel.	
SY	SY1	The visual interface of the AI system presents real-time navigation and payment updates with high clarity.	Meng and Zhu (2022)
	SY2	I feel a strong sense of operational control when interacting with the dynamic menus of the AI platform.	
	SY3	The sensory feedback provided by the interface reduces my technical confusion during financial transactions.	
	SY4	The data transmission processes shown on the screen are highly transparent and secure.	
JX	JX1	The overall layout and typography of the AI user interface exhibit high professional design integrity.	Cao (2013), Karahanna et al. (1999), and Aladwani and Palvia (2002)
	JX2	The structural composition of the digital gateway reflects rigorous industrial standards.	
	JX3	The visual organization of the system interface appears well-ordered and cohesive.	
	JX4	The technical architecture of the platform looks reliable enough to manage micro-financial operational risks.	
QW	QW1	I expected the AI system to provide a zero-error environment for my on-board transaction tracking.	Lin et al. (2005), Hsu et al. (2006), Kang et al. (2009), Dai and Liu (2015), and Zhan and Yan (2014)
	QW2	I expected the platform to neutralize potential data privacy risks and fraud vulnerabilities effectively.	
	QW3	I expected the system backend to maintain robust compliance control during cross-border transit commerce.	
QC	QC1	The actual performance of the AI platform in safeguarding my transactions confirmed my initial expectations.	Lin et al. (2005), Hsu et al. (2006), Kang et al. (2009), Dai and Liu (2015), and Zhan and Yan (2014)
	QC2	The secure experience provided by the system exceeded my baseline psychological security requirements.	
	QC3	The interface feedback successfully confirmed that my personal data and assets were fully protected.	
MY	MY1	I am thoroughly satisfied with the safety performance of this cruise intelligent guidance system.	Oliver (1981), Churchill and Surprenant (1982), and Patterson et al. (1997)
	MY2	I can completely trust this automated gateway to handle my financial payments in network-restricted environments.	
	MY3	Based on my usage experience, I have a high level of long-term commitment and trust toward this complex mobile economy system.	

### 3.3 Questionnaire survey

China’s inaugural independently constructed mega cruise vessel, Adora Magic City, was chosen as the empirical setting for this investigation. The Adora Magic City spans approximately 34,600 square meters across 15 decks, featuring 2,125 cabins and a maximum capacity of 5,246 passengers. Its maiden voyage itinerary covers Shanghai, Jeju, Nagasaki, and Fukuoka, offering a 6-night, 7-day journey filled with diverse onboard experiences. The ship combines meticulous craftsmanship in hull design and spatial planning with innovative integration of themed dining and entertainment programs, creating a unique East–West cultural and tourism fusion journey aboard the Adora Magic City.

The itinerary of the Adora Magic City typically spans 5 days: the first day is the boarding day, the second day is a sea cruise day, the third and fourth days are shore excursion days, and the fifth day is the disembarkation day. For this study, on-site surveys were conducted at each of these key time points. The research focused on passengers participating in the 5-day short cruise journey. In April 2024, the research team boarded the Adora Magic City to conduct a questionnaire survey themed “Perceived Experience of the Cruise Intelligent Guidance System.” Participants were recruited in public areas such as restaurants, theaters, shopping zones, water parks, gyms, and basketball courts. Participants were required to have spent at least 20 min in the designated spaces before completing the questionnaire, which aimed to investigate their wayfinding experiences. This temporal threshold was strictly implemented to ensure that participants had accumulated high-frequency, in-depth sensory interaction flows with the AI guidance platform within the complex physical environments, and had executed substantive micro-financial digital payments or navigation decisions, thereby guaranteeing the authenticity, validity, and objective traceability of the survey data. A semi-structured survey method was employed in this study. The survey process encouraged active participation, and participants received a commemorative souvenir as an incentive. Data collection took place over a six-day period between April 30 and May 5, 2024. During this interval, 350 survey instruments were issued, of which 329 were deemed usable, corresponding to an effective return proportion of 94 percent. Participation occurred on a voluntary basis, responses were recorded without personal identifiers, and informed consent was obtained from all respondents before administration of the questionnaire.

This research was implemented through an anonymous survey-based approach and did not involve any form of experimental intervention on human subjects. Participant involvement was confined to voluntary social data provision, with no collection of identifiable personal information. All procedures adhered to applicable institutional standards and regulatory requirements, and the study protocol was reviewed and authorized by the author’s affiliated organization. Prior to participation, respondents were fully informed of the study purpose and provided consent in line with internationally recognized ethical principles, including those articulated in the Declaration of Helsinki.

Furthermore, in February 2023, relevant authorities in China issued updated administrative provisions governing human-related biomedical research. According to Article 32 of these provisions, studies relying exclusively on anonymized human data, posing no risk to participants, and excluding sensitive or commercial information may be exempt from formal ethical review. As this investigation solely utilized de-identified survey data for academic analysis, it meets the stated exemption criteria and does not raise ethical concerns.

Table 2 clearly presents the demographic characteristics and intelligent system usage behaviors of the 329 valid respondents in this empirical study. The gender distribution among the participants is relatively balanced, with females (54.10%) slightly outnumbering males (45.90%). In terms of age structure, the respondents are predominantly concentrated in the young and middle-aged groups between 26 and 45 years old, collectively accounting for over 63% and establishing them as the core demographic for digital interactions and consumption. Regarding the usage behavior of the AI-guided system, only 20.67% of the passengers are first-time users, whereas up to 79.33% exhibit occasional or frequent usage habits. Overall, the data distribution is reasonable, which not only highly conforms to the authentic profile of passengers on modern mega cruise ships but also provides a robustly representative sample foundation for the subsequent analysis of secure sensory interactions and trust building between passengers and the system.

**Table 2:** Respondent demographics and AI guidance system usage characteristics.

Demographic variable	Category	Frequency (n)	Percentage (%)
Gender	Male	151	45.9
	Female	178	54.1
Age	≤25 years old	43	13.07
	26–35 years old	118	35.87
	36–45 years old	92	27.96
	46–55 years old	51	15.5
	>55 years old	25	7.6
AI system usage frequency	First-time user	68	20.67
	Occasional user (1–2 times/day)	142	43.16
	Frequent user (≥ 3 times/day)	119	36.17

## 4 Data analysis

For data derived from standardized instruments, analysis begins with a comprehensive evaluation of the measurement properties, encompassing consistency, convergent accuracy, discriminant clarity, and construct integrity. This step focuses on verifying the suitability of the measurement structure within the proposed model based on observed data. Once these criteria are satisfactorily met, subsequent procedures include assessing potential common method effects and examining inter-variable associations. If no significant common method bias is detected, a structural model is constructed for subsequent hypothesis testing. The software used for these analyses are SPSS 26.0 and AMOS 27.

To facilitate data analysis, the study defines the variables as follows: Contextual-Technology Fit (RK), Secured Sensory Interaction (SY), Perceived Architectural Quality (JX), Security Expectations (QW), Reassurance Confirmation level (QC), and Trust-Satisfaction (MY).

### 4.1 Reliability analysis

Scale reliability reflects the degree to which measurement results remain consistent and dependable. In this study, internal reliability was examined using Cronbach’s *α* statistics computed with SPSS 26.0. In empirical research, coefficient values exceeding 0.70 are generally regarded as meeting acceptable standards. The analysis yielded an overall α value of 0.907 for the dataset, while reliability indices for individual constructs are reported in Table 3. As indicated, all latent constructs achieved α coefficients above the recommended threshold, confirming strong internal consistency and supporting the suitability of the data for subsequent analytical procedures.

**Table 3:** Internal consistency reliability of the measurement constructs.

Measurand	Number of questions	Cronbach’s α
RK	5	0.848
SY	4	0.911
JX	4	0.898
QW	3	0.869
QC	3	0.859
MY	3	0.849

### 4.2 Validity analysis

**Figure 2:**
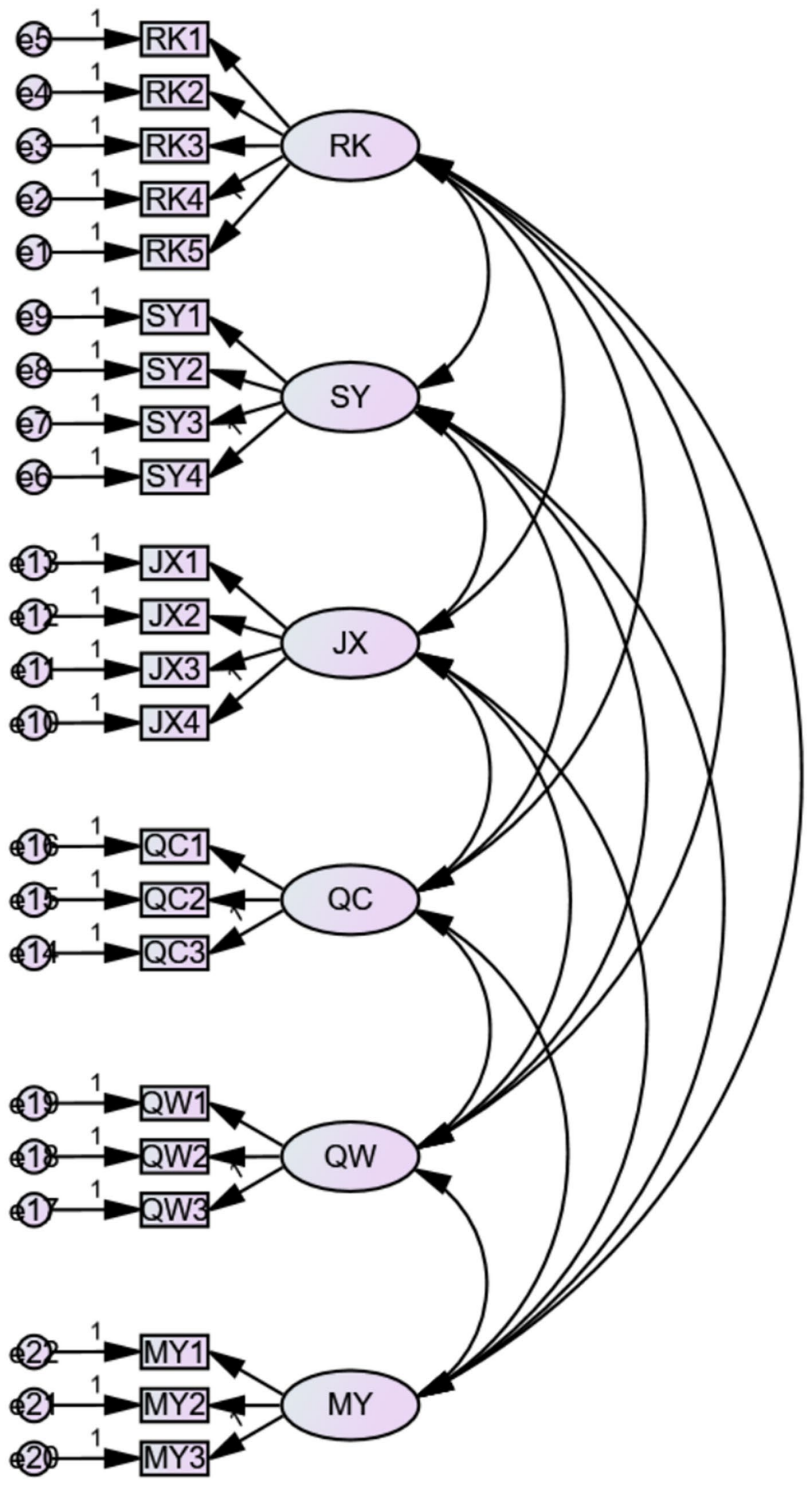
Confirmatory factor analysis model for the six latent constructs.

Measurement validity reflects how effectively an instrument represents the theoretical concept it is intended to assess, encompassing aspects such as convergence, distinction among constructs, and overall structural coherence. Using SPSS 26.0, the dataset was subjected to preliminary diagnostic testing, producing a Kaiser–Meyer–Olkin index of 0.893 and a statistically significant Bartlett’s sphericity statistic (χ^2^ ≈ 4558.441), indicating suitability for factor-based analysis. These outcomes suggest that the data demonstrate an acceptable level of validity. Subsequently, the proposed structural framework was specified in AMOS 27 (see Figure 2), and exploratory factor analysis was performed to examine the underlying factor structure.

Prior to interpreting the results of the exploratory factor analysis, it is necessary to evaluate construct-level adequacy to determine whether the proposed structure is empirically supported. Should the specified configuration fail to align with the observed data, the assumed relationships among variables must be reconsidered and reformulated. Model adequacy is assessed using a combination of goodness-of-fit indicators that reflect absolute, relative, and parsimonious performance. An acceptable fit is indicated when the chi-square to degrees of freedom ratio ranges between 1 and 3, while a root mean square error of approximation below 0.05 further supports structural appropriateness. In addition, comparative measures of relative fit, including the Incremental Fit Index, Comparative Fit Index, and Tucker–Lewis Index, are expected to surpass the 0.90 criterion. Measures of model simplicity, namely the Parsimony Goodness-of-Fit Index and Parsimony Normed Fit Index, should each exceed 0.50. As reported in Table 4, all evaluated indices satisfy these benchmark values, confirming that the model demonstrates an adequate level of fit.

**Table 4:** Goodness-of-fit indices for the measurement model.

Main fitting indicators	Standard value range	Value of this model
X^2^/df	1–3	1.413
RMSEA	<0.05	0.034
NFI	>0.9	0.941
IFI	>0.9	0.982
TLI	>0.9	0.979
CFI	>0.9	0.982
PCFI	>0.5	0.825
PNFI	>0.5	0.790

Convergent validity is commonly assessed through indicators such as Composite Reliability and Average Variance Extracted. As summarized in Table 5, the evaluation outcomes for each construct show that all measurement items exhibit standardized loadings greater than 0.70 on their corresponding factors, confirming appropriate alignment between indicators and theoretical dimensions. Moreover, the extracted variance for each construct exceeds the 0.50 threshold, while reliability coefficients surpass 0.80, collectively indicating adequate convergence and robust internal coherence.

**Table 5:** Standardized factor loadings, composite reliability, and average variance extracted for convergent validity.

Path			Standard factor loadings	CR	AVE
RK1	<−--	RK	0.741	0.850	0.531
RK2	<−--	RK	0.715		
RK3	<−--	RK	0.738		
RK4	<−--	RK	0.742		
RK5	<−--	RK	0.708		
SY1	<−--	SY	0.837	0.912	0.721
SY2	<−--	SY	0.832		
SY3	<−--	SY	0.850		
SY4	<−--	SY	0.876		
JX1	<−--	JX	0.823	0.899	0.689
JX2	<−--	JX	0.856		
JX3	<−--	JX	0.828		
JX4	<−--	JX	0.812		
QC1	<−--	QC	0.826	0.860	0.672
QC2	<−--	QC	0.804		
QC3	<−--	QC	0.829		
QW1	<−--	QW	0.851	0.869	0.689
QW2	<−--	QW	0.803		
QW3	<−--	QW	0.836		
MY1	<−--	MY	0.790	0.849	0.653
MY2	<−--	MY	0.838		
MY3	<−--	MY	0.795		

Following the establishment of measurement reliability and convergent adequacy, the distinctiveness of the constructs was examined through discriminant assessment. This evaluation typically involves contrasting the square roots of the Average Variance Extracted for each construct with the inter-construct correlation values. Adequate separation among variables is indicated when the extracted variance square root for a given construct surpasses its correlations with other constructs. As presented in Table 6, this condition is met across all latent dimensions, demonstrating clear differentiation among constructs and confirming acceptable discriminant soundness of the dataset.

**Table 6:** Discriminant validity matrix for the six latent constructs.

Construct	RK	SY	JX	QC	QW	MY
RK	**0.729**					
SY	0.371	**0.849**				
JX	0.32	0.357	**0.830**			
QC	0.399	0.392	0.439	**0.820**		
QW	0.338	0.356	0.313	0.268	**0.830**	
MY	0.499	0.582	0.492	0.582	0.459	**0.808**

Diagonal elements (bolded) represent the square roots of the Average Variance Extracted (AVE) for each latent construct; off-diagonal elements represent the Pearson correlation coefficients between constructs.

### 4.3 Common method bias test

**Figure 3:**
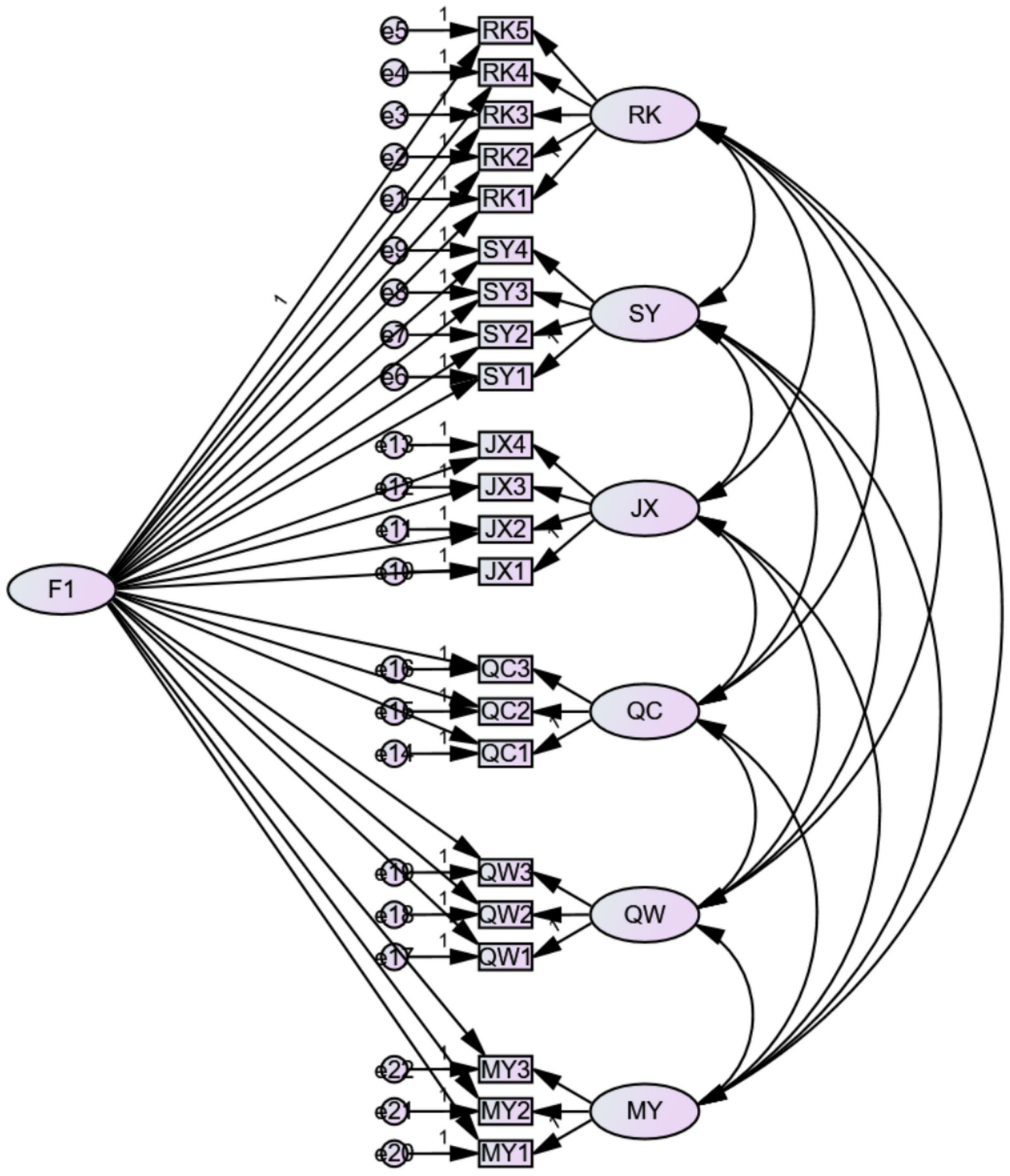
Common method bias assessment using an unmeasured latent method factor.

Common method bias describes spurious covariance between independent and dependent constructs that arises from shared measurement sources, evaluators, contextual settings, or item characteristics rather than true theoretical relationships. In addition to implementing procedural remedies such as anonymous data collection, this study applied the unmeasured latent method factor technique to statistically diagnose potential bias. Specifically, an additional method-related latent construct was introduced into the confirmatory factor analysis framework, following the approach proposed by Lian et al. (2018), as depicted in Figure 3. A revised measurement model was specified by incorporating a latent factor (F17) that loaded on all observed indicators, and its performance was compared against the original CFA model used in the validity assessment.

Comparative analysis of model fit statistics between the two specifications yielded the following differences: Δχ^2^/df = 0.111, ΔCFI = 0.006, ΔIFI = 0.006, ΔTLI = 0.005, ΔRMSEA = 0.005, and ΔSRMR = 0.0102. According to widely accepted academic thresholds in structural equation modeling, a variance of less than 0.02 in these indices demonstrates that the common method factor does not significantly improve the model fit. Therefore, these outcomes directly confirm that common method bias (CMB) does not pose a substantial threat to the validity of the data or the subsequent hypothesis testing in this study. The inclusion of the method factor did not result in a meaningful enhancement of model fit. The inclusion of the method factor did not result in a meaningful enhancement of model fit. Moreover, the observed changes in RMSEA and SRMR remained well below 0.05, and the variations in CFI and TLI were under 0.10, indicating that common method effects are unlikely to pose a serious threat to the validity of the findings.

### 4.4 Model fit test

**Figure 4:**
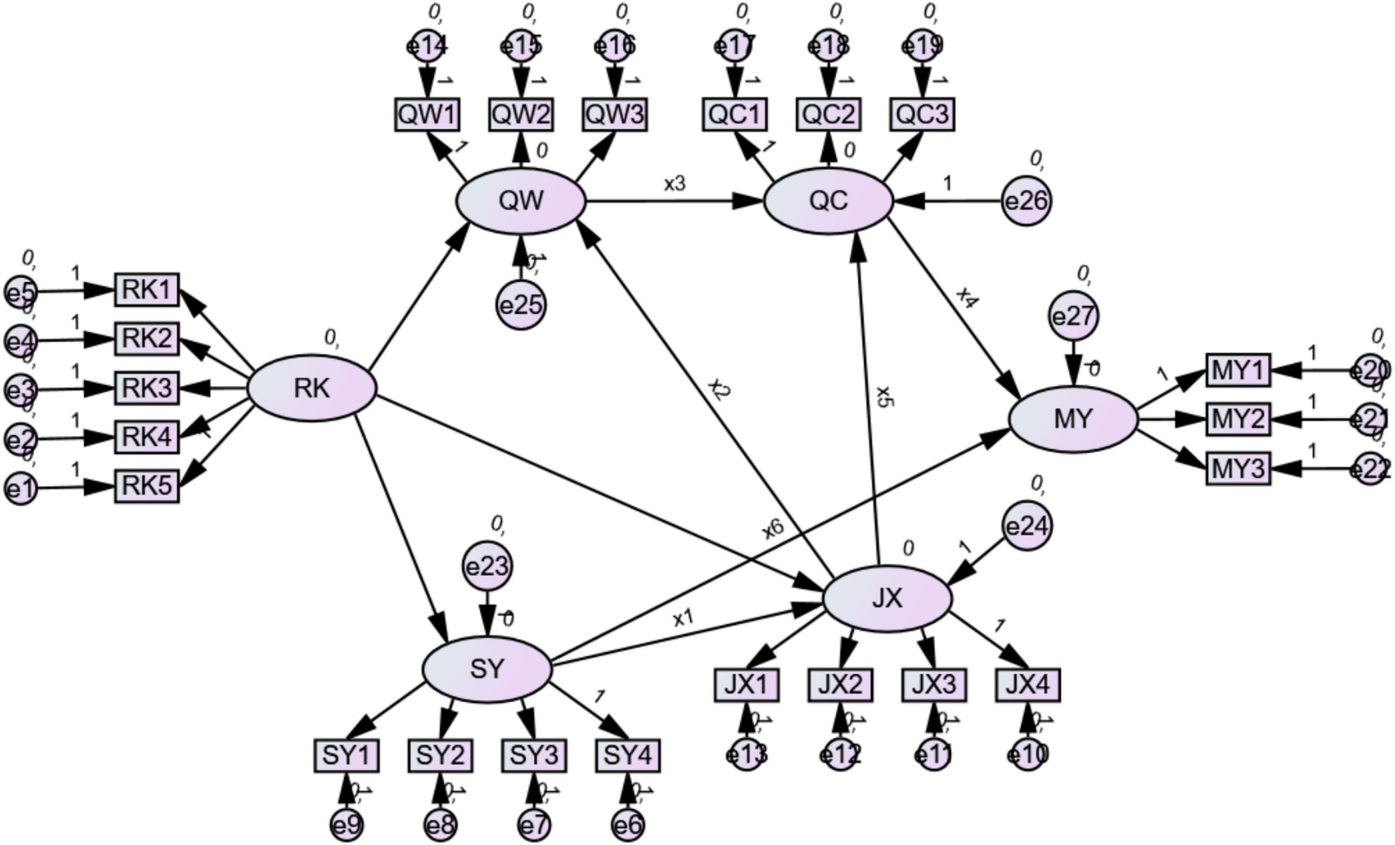
Standardized structural model showing significant pathways to trust-satisfaction.

The proposed structural framework was specified using AMOS 27, in which MY served as the outcome construct, while all other latent variables were modeled as predictors. Directional paths were defined exclusively from the explanatory constructs toward MY. After configuring all hypothesized paths, the survey data were imported for confirmatory factor analysis (CFA), and the structural relationships between variables are illustrated in Figure 4. Maximum Likelihood Estimation (ML) was employed for path analysis. Structural validity was first assessed, followed by model fit evaluation.

The results summarized in Table 7 demonstrate that the principal goodness-of-fit statistics, such as the chi-square to degrees of freedom ratio, the root mean square error of approximation, and the comparative fit index, all fall within accepted benchmark ranges, confirming that the structural model corresponds well with the observed data.

**Table 7:** Goodness-of-fit indices for the structural model.

Main fitting indicators	Standard value range	Value of this model
X^2^/df	1–3	1.780
RMSEA	<0.05	0.047
NFI	>0.9	0.924
IFI	>0.9	0.912
TLI	>0.9	0.959
CFI	>0.9	0.965
PCFI	>0.5	0.835
PNFI	>0.5	0.800

According to the path test results in Table 8, all nine paths in the hypothesized model exhibited positive and significant effects (all *p*-values were less than 0.05; in AMOS, p-values below 0.001 are denoted as ***). Specifically:

**Table 8:** Standardized path coefficients and hypothesis-testing results.

Path	Standardized path coefficients	**S. E.**	**C. R.**	**P**
RK → SY	0.392	0.078	6.267	***
RK → JX	0.217	0.07	3.387	***
RK → QW	0.28	0.083	4.295	***
SY → JX	0.295	0.054	4.783	***
SY → MY	0.448	0.041	7.822	***
JX → QW	0.226	0.071	3.684	***
JX → QC	0.414	0.059	6.626	***
QW → QC	0.167	0.048	2.796	0.005
QC → MY	0.451	0.053	7.577	***

RK positively and significantly influenced SY, JX, and QW.

SY positively and significantly influenced JX and MY.

JX positively and significantly influenced QW and QC.

QW positively and significantly influenced QC.

QC positively and significantly influenced MY.

The magnitude of these effects can be interpreted based on their respective regression coefficients.

### 4.5 Mediation effect test

In the constructed model, potential mediation relationships exist between multiple independent variables and the dependent variable (MY). For example, the variable SY influences MY through JX and QC. While correlation analysis reveals covariation trends among variables, further mediation testing is required to clarify how these variables interact, whether mediation effects exist, and their magnitude.

Using the bias-corrected nonparametric percentile Bootstrap method (with 5,000 repetitions), mediation tests were conducted. The results are shown in Table 9.

**Table 9:** Bootstrap mediation effects for direct, indirect, and total pathways.

Effect	Effect value	SE	Bias-corrected 95%CI			Percentile 95%CI		
			Lower	Upper	*P*	Lower	Upper	*P*
stdIndA1	0.055	0.016	0.030	0.080	0.019	0.032	0.086	0.010
stdIndA2	0.448	0.053	0.335	0.519	0.016	0.354	0.524	0.010
stdIndA3	0.503	0.051	0.404	0.572	0.019	0.408	0.580	0.010
stdIndA4	0.005	0.003	0.001	0.012	0.011	0.001	0.010	0.016
stdIndA5	0.453	0.053	0.346	0.522	0.016	0.356	0.528	0.010
stdIndA6	0.038	0.017	0.018	0.072	0.005	0.011	0.068	0.016
stdIndA7	0.414	0.058	0.296	0.491	0.020	0.314	0.502	0.010
stdIndA8	0.452	0.054	0.344	0.525	0.018	0.350	0.528	0.010

SY = Secured Sensory Interaction; JX = Perceived Architectural Quality; QW = Security Expectations; QC = Reassurance Confirmation; MY = Trust-Satisfaction. stdIndA1 represents the specific mediation path: SY → JX → QC → MY. stdIndA2 represents the direct path: SY → MY. stdIndA3 represents the total effect of the mediation model: SY → JX → QC → MY (including the direct path SY → MY). stdIndA4 represents the specific chain mediation path: SY → JX → QW → QC → MY. stdIndA5 represents the total effect of the chain mediation model: SY → JX → QW → QC → MY (including the direct path SY → MY). stdIndA6 represents the specific mediation path: JX → QW → QC. stdIndA7 represents the direct path: JX → QC. stdIndA8 represents the total effect of the framework: JX → QW → QC.

#### 4.5.1 Standardized mediation effect test results

The direct effect of SY on MY is 0.448, with a 95% confidence interval (CI) of [0.335, 0.519], excluding 0, indicating a statistically significant direct effect.

The indirect effect of SY → JX → QC → MY is 0.055 (95% CI: [0.030, 0.080]), excluding 0, confirming a significant mediation path. The total effect of this mediation model (SY → JX → QC → MY) is 0.503 (95% CI: [0.404, 0.572]), demonstrating overall significance.

Another mediation path (SY → JX → QW → QC → MY) shows an indirect effect of 0.005 (95% CI: [0.001, 0.012]), with a total effect of 0.453 (95% CI: [0.346, 0.522]), both excluding 0, further supporting the existence of mediation.

#### 4.5.2 Exploration of additional mediation relationships

The relationship among JX, QW, and QC was tested. The indirect effect of JX → QW → QC is 0.038 (95% CI: [0.018, 0.072]), excluding 0, indicating a significant mediation.

The direct effect of JX → QC is 0.414 (95% CI: [0.296, 0.491]), and the total effect of the mediation model (JX → QW → QC) is 0.452 (95% CI: [0.344, 0.522]), both statistically significant.

These results confirm the presence of complex mediation pathways in the model, where variables like JX and Reassurance Confirmation act as critical intermediaries in shaping user MY.

## 5 Discussion

The empirical results of this study validate the structural relationships within the integrated framework of RK and Reassurance Confirmation. The model effectively explains how an AI-driven guidance system functions as a micro-risk management mechanism that mitigates behavioral and financial transactional risks in the complex economy of a mega cruise ship. The statistical analysis demonstrates that RK exerts a strong positive influence on both SY and JX.

### 5.1 The driving role of contextual-technology fit in alleviating operational risks

The statistical analysis demonstrates that RK exerts a strong positive influence on both SY and JX. The task-technology fit theory emphasizes that the alignment between technological attributes and individual task requirements is the core mechanism for improving operational performance and acceptance (Goodhue and Thompson, 1995). This finding underscores that in a closed and dense economic environment the alignment between AI navigational assistance and specific environmental risks is foundational. A mega cruise liner is essentially a mobile economic system involving cross-border clearance and high-frequency consumption. In this complex context effective information seeking tools and technology fit can significantly reduce uncertainty in the decision-making environment (D’Ambra and Rice, 2004).

If the technological features of the AI guidance system fail to precisely match the spatial navigation tasks of passengers it will trigger severe decision-making errors and financial risks. Environmental signage and wayfinding design exert a decisive intervention effect on individual behavioral trajectories within complex public transit hub systems (Gray et al., 2021). When the system exhibits a high level of contextual fit it can resolve the micro-risks brought by spatial uncertainty through precise information flow distribution. Underground spaces or highly enclosed architectural volumes often deprive individuals of their natural sense of direction and induce psychological panic (Jasińska, 2023). The high fit of the system not only improves the efficiency of physical space circulation but also reduces passengers’ anxiety regarding system failures at a psychological level. This indicates that users’ perceived technology fit depends on the extent to which the AI-guided platform matches the specific security needs of the scenario (Dishaw and Strong, 1999).

### 5.2 Mechanisms of perceived architectural quality in trust building

The results indicate that JX is a critical node connecting objective technological performance with user psychological confirmation. This variable significantly influences users’ QW and Reassurance Confirmation (QC). A user’s subjective perception of the overall quality of a network or digital system directly determines their subsequent interaction willingness and trust depth (Aladwani and Palvia, 2002). The statistical validation of this path is crucial for understanding the visual order within digital financial systems.

The empirical results indicate that the professional visual presentation of the AI interface (JX) serves as the primary basis for passengers to confirm their initial QW. In high-risk perception environments like mega cruise ships, a user’s subjective evaluation of layout typography and interface quality directly validates their baseline safety anticipations. This highly normalized visual order transmits a psychological signal of a robust and fully compliant backend system, effectively transforming immediate visual cues into a form of structural assurance or institutional trust similar to that found in modern electronic marketplaces. Ultimately, this visual trust mechanism successfully mitigates financial anxiety and privacy concerns within enclosed, network-restricted spaces. By eliminating operational panic, it allows passengers to smoothly shift their attention from defensive vigilance to normal consumption experiences, achieving a complete logical loop of security reassurance.

A high-quality AI interaction gateway will continuously update passengers’ evaluation criteria for the entire micro-economic system. The system must constantly surpass users’ psychological baselines through continuous high-quality feedback and neutralize potential systemic risk perceptions in real time.

### 5.3 Direct contributions of secured sensory interaction to trust-satisfaction and system security

SY has a direct and highly significant driving effect on overall MY. Customer satisfaction is a multi-dimensional psychological state directly determined by actual experience performance and its core lies in whether expectations are met (Oliver, 1981). This proves from a data perspective that the interaction experience itself is a highly central risk-control value proposition. In the specific complex consumption scenarios of cruise ships the absolute smoothness of interaction and data transparency directly determine passengers’ psychological sense of gain (Churchill and Surprenant, 1982). The critical factors determining final customer satisfaction are often embedded within the subtlest service encounter processes (Patterson et al., 1997).

When passengers obtain clear and deterministic feedback while interacting with the AI system they develop a sense of mastery of being in a completely controlled state. This psychological state can significantly reduce behavioral disorders and impulsive consumption risks triggered by environmental complexity. Empirical research on the acceptance of healthcare wearable devices also indicates that in fields involving personal or high privacy security the reliability and intuitiveness of sensory interfaces are paramount (Wang et al., 2020). SY not only directly enhances terminal satisfaction but also deeply reshapes users’ cognitive evaluation systems. Interface-level visual cues, including transaction-state indicators, payment confirmation feedback, and security prompts, can significantly affect users’ perceived safety boundaries and confidence in the system.

Passengers’ judgment of digital system architectural quality is not static but dynamically formed during repeated spatial navigation interactions. Frequent and highly successful interaction processes deepen passengers’ recognition of AI professionalism thereby thoroughly strengthening the baseline trust orientation toward the entire mobile financial system. In a macro perspective SY can function as a crowd warning and traffic diversion tool. Through intuitive user interfaces the system optimizes overall crowd distribution and ensures absolute behavioral compliance at all financial payment nodes. This is the exact concrete manifestation of cutting-edge AI technology empowering complex system security.

### 5.4 Efficacy analysis of chain mediation and the reassurance circuit

This study validates multiple complex mediation paths through rigorous statistical testing. Among them the chain effect triggered from SY to JX which then leads to reassurance confirmation and ultimately guides overall satisfaction is the most prominent. Merging traditional task-technology fit theory with expectation confirmation theory can more comprehensively and deeply explain individual continuous behavioral patterns and performance in complex online environments (Ayyoub et al., 2023). This rigorous logical circuit completely reveals the full life cycle of security trust generation.

This generation process begins with basic physical screen interactions passes through cognitive evaluation of architectural quality by the brain precisely triggers the psychological confirmation of security and finally consolidates into irreversible emotional trust. In the field of e-commerce services users’ continuance intention heavily relies on the actual degree to which initial expectations are confirmed by the system (Bhattacherjee, 2001). Predicting electronic service continuance combined with a decomposed theory of planned behavior indicates that intention formation is a multi-stage belief evolution process (Hsu and Chiu, 2004). This fully demonstrates that AI risk control in complex financial systems must not rely solely on a single functional algorithm output but must construct a completely closed-loop psychological and emotional feedback system.

Long-term longitudinal studies on online consumer behavior show that users’ initial beliefs and systemic risk perceptions will undergo structural evolution as operational experience accumulates (Hsu et al., 2006). The existence of multiple mediation effects means that the reassurance confirmation variable plays a central psychological regulatory role in the entire model. The mediation results indicate that user trust is not generated by back-end technical capability alone; it depends on whether the AI interface can translate system performance into clear, interpretable, and reassuring interaction feedback. Even if the backend digital system possesses strong technical computing power its final trust and satisfaction will face a precipitous drop if it fails to achieve confirmation at the human psychological level.

In the study of information technology adoption across time actual usage experience holds a decisive power in reconstructing user beliefs (Karahanna et al., 1999). The AI system acts as a crucial psychological mediator here perfectly translating obscure technical parameters into human-perceptible security assurance signals. This data-driven chain effect analysis provides solid theoretical guidance for the digital upgrade of all complex systems. Top-level system design should fully consider this progressive trust-building logic ensuring that each execution node of the underlying code strictly serves the higher-level goal of group security confirmation.

### 5.5 Theoretical contributions to AI financial risk management

This study performs a brand-new contextual integration and interdisciplinary expansion of multiple traditional behavioral theoretical frameworks. The research creatively proposes the concept of RK expanding the theoretical domain of technology fit from pure tool efficiency to comprehensive dimensions including environmental safety and systemic risk perception. AI-driven customer interaction adoption behaviors are gradually reshaping the theoretical boundaries of traditional service marketing and corporate risk management (Vafaei-Zadeh et al., 2025). The establishment of this model effectively responds to the strong academic call to re-examine the boundary conditions of classic technology acceptance models in complex innovation scenarios.

This study explicitly validates the unique theoretical value of reassurance confirmation (QC) as a core mediating variable. In the digital city context involving the reshaping of underlying data rights and algorithm governance individuals’ psychological confirmation of information security is the core shield for society against systemic collapse risks (Thornton, 2022). This core finding provides an important new variable dimension for future research regarding the deep intervention of AI in complex financial systems.

This research further clarifies how AI systems create value in micro-economic risk control within complex spatial environments. The contribution of interface architecture in this study is not understood as public art, place-making, or spatial narrative, but as a functional mechanism for financial risk communication. In a closed-loop mobile economy, the interface must help users distinguish whether a payment has been initiated, synchronized, verified, completed, or interrupted. Clear visual hierarchy, consistent feedback, and interpretable security prompts therefore serve as front-end signals of back-end financial protection. Prior research on mobile payment services has shown that perceived control and interface design features significantly shape users’ perceived security and continuous use intention (Zhang J. et al., 2019). In parallel, research on explainable AI in finance emphasizes that transparency and interpretability are critical for converting algorithmic risk-control capability into stakeholder trust (Weber et al., 2024). By empirically validating the effects of Secured Sensory Interaction and Perceived Architectural Quality, this study demonstrates that AI-driven financial risk management operates through both algorithmic control and psychological reassurance. This theoretical shift helps explain how users convert interaction fluency and visual order into perceived security, trust confirmation, and satisfaction during high-frequency micro-payment activities.

### 5.6 Design optimization and management implications

For international cruise operators and underlying digital system architecture designers this research provides an exceptionally clear path for commercial optimization. The focus of frontend design work should immediately shift from simple physical navigation tool development to deep secured interaction experience reshaping.

In the early stages of user interface development, visual structural elements including payment environment safety prompts and compliance operation guidance must be thoroughly integrated to reshape the secure interaction experience. Managers should establish high-frequency, dynamic expectation management mechanisms within the platform. By tracking real-time user interaction metrics and operational pause data across different physical voyage segments, the AI system can intelligently adjust the intervention intensity of its underlying security strategies. Furthermore, because minor operational disconnections or interface freezes can immediately trigger user anxieties regarding fund theft or privacy leakage, the technical development team must conduct rigorous system boundary pressure tests. The intelligent guidance infrastructure must be engineered to provide smooth localized credential verification and offline risk-avoiding path planning services in deep-sea environments with zero network signals, thereby ensuring absolute operational security and continuous trust confirmation.

Enterprises can treat the intelligent digital guidance system as a core strategic component to maintain international brand reputation and reduce systemic operational risks from the source. Establishing a comprehensive AI governance system with highly matched scenarios and extremely efficient confirmation feedback can effectively and continuously enhance passenger loyalty and provide the lowest-level security barrier for the sustainable profitable development of micro-economic entities.

### 5.7 Research limitations and future directions

Although this study provides a highly unique and self-consistent theoretical perspective certain limitations objectively exist and must be overcome in subsequent inquiries. The empirical data for this survey stems entirely from a single domestic mega cruise liner and this sample homogeneity objectively limits the generalizability of the conclusions across global routes and diverse cultural backgrounds. Future large-scale research should actively conduct comparative analyses across brands and regions to validate the cross-environmental stability of this trust-building model. Users’ continuous usage behavior toward mobile applications is often structurally constrained by personal habits and long-term platform dependencies over time (Tam et al., 2020).

This study utilizes cross-sectional data from a specific point in time which mathematically cannot capture the full picture of the dynamic evolution of human trust. In-depth research on mobile app continuance intentions strongly recommends that scholars adopt longitudinal tracking survey designs to accurately reveal the objective laws of user attitudes and trust changing over time.

Emerging core technologies such as generative AI and large language models are profoundly changing the user acceptance logic of complex information systems. Future research could explore how to utilize these advanced tools to implement intelligent real-time risk alerts and natural-language-based multimodal digital-human guidance. This exploration will dynamically extend the proposed RK model into the generative AI ecosystem, transforming static system parameters into adaptive risk governance mechanisms. These underlying technological breakthroughs will inevitably assist humanity in building a digital financial ecosystem defense framework with stronger self-repair resilience.

## 6 Conclusion

This study integrates the task-technology fit theory with the expectation confirmation theory to construct an explanatory model for user satisfaction and system security regarding artificial intelligence guidance systems on mega cruise ships. Through an empirical analysis of data collected from China’s first domestically built mega cruise vessel, the research systematically examines the risk-control pathways and trust-building mechanisms of intelligent systems within complex micro-economic environments.

The empirical results demonstrate that contextual-technology fit is a critical antecedent driving the secure operation of the system. When the technological features of the AI guidance system align deeply with the spatial navigation tasks of passengers, it significantly enhances the quality of secured sensory interactions and strengthens passengers’ perception of the system architectural quality. This process effectively mitigates behavioral uncertainties within highly dense and enclosed spaces, reducing decision-making errors and potential financial transaction risks from the source.

The study further confirms the critical mediating roles of perceived architectural quality and reassurance confirmation in the process of trust building. The professional visual presentation and efficient interactive feedback of the system transmit psychological signals of backend stability and regulatory compliance to end users. The psychological confirmation of security triggered by this visual order successfully transforms passengers’ defensive vigilance into baseline trust toward the entire mobile economic system, ultimately serving as a decisive force that determines overall trust-satisfaction.

The theoretical contribution of this study lies in extending the boundary conditions of classic technology acceptance models. By upgrading the scope of technology fit from pure tool efficiency to a comprehensive dimension encompassing spatial safety and systemic risk perception, this research provides a novel behavioral perspective for financial technology governance in complex closed-loop environments. In terms of managerial practice, the findings offer a clear optimization path for cruise operators and system designers. The focus of top-level design should shift from simple functional development to deep secured interaction experience reshaping, establishing a comprehensive algorithm governance system with highly matched scenarios and efficient feedback to ensure both the sustainable profitability of micro-economic entities and systemic risk control.

## Data Availability

The raw data supporting the conclusions of this article will be made available by the authors, without undue reservation.
